# Supplementary material for: The MetaInvert soil invertebrate genome resource provides insights into below-ground biodiversity and evolution
Source: Commun Biol. 2023 Dec 8;6:1241. doi: 10.1038/s42003-023-05621-4 (PMC10709333; doi:10.1038/s42003-023-05621-4)
Supplement: Supplementary file 2 — Supplementary Information [file 42003_2023_5621_MOESM2_ESM.pdf]

Supplementary Information

Supp. Fig. 1. Maximum likelihood phylogenetic tree based on an alignment of 141 metazoan BUSCO genes of the 232 soil invertebrates sequenced in this work (coloured branches), and 118 NCBI RefSeq (grey branches), representing four phyla. Families are annotated on the tree to show phylogenetic breadth. Dots highlight nodes with over 75% bootstrap support.

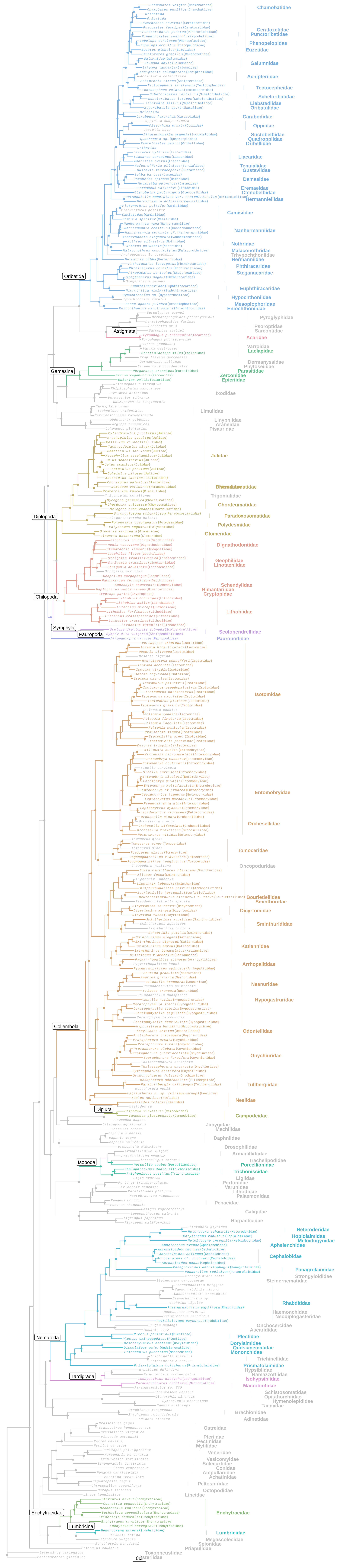

Chamobatidae
Ceratozetidae
Punctoribatidae
Phenopelopidae
Euzetidae
Galumnidae
Achipteriidae
Tectocepheidae
Scheloribatidae
Liebstadiidae
Oribatulidae
Carabodidae
Oppiidae
Suctobelbidae
Quadroppiidae
Oribellidae
Liacaridae
Tenuialidae
Gustaviidae
Damaeidae
Eremaeidae
Ctenobelbidae
Hermanniellidae
Camisiidae
Nanhermanniidae
Nothridae
Malaconothridae
Trhypochthoniidae
Hermanniidae
Phthiracaridae
Steganacaridae
Euphthiracaridae
Hypochthoniidae
Mesoplophoridae
Enjochthoniidae
Oribatida
Pyroglyphidae
Psoroptidae
Sarcoptidae
Astigmata
Acaridae
Varroidae
Laelapidae
Dermanyssidae
Phytoseiidae
Parasitidae
Zerconidae
Epicriidae
Gamasina
Ixodidae
Limulidae
Linyphiidae
Araneidae
Pisauridae
Julidae
Blaniulidae
Nemasomatidae
Trigoniulidae
Chordeumatidae
Paradoxosomatidae
Polydesmidae
Glomeridae
Diplopoda
Dignathodontidae
Geophilidae
Linotaeniidae
Schendylidae
Himantariidae
Cryptopidae
Chilopoda
Lithobiidae
Scolopendrellidae
Pauropodidae
Symphyla
Pauropoda
Isotomidae
Entomobryidae
Orchesellidae
Tomoceridae
Oncopoduridae
Bourletiellidae
Sminthuridae
Dicyrtomidae
Sminthurididae
Katiannidae
Arrhopalitidae
Neanuridae
Hypogastruridae
Odontellidae
Onychiuridae
Tullbergiidae
Neelidae
Collembola
Diplura
Campodeidae
Japygidae
Machilidae
Daphniidae
Drosophilidae
Armadillidiidae
Trachelipodidae
Porcellionidae
Trichoniscidae
Isopoda
Ligiidae
Portunidae
Varunidae
Lithodidae
Palaemonidae
Penaeidae
Caligidae
Harpacticidae
Heteroderidae
Hoplolaimidae
Meloidogynidae
Aphelenchidae
Cephalobidae
Panagrolaimidae
Strongyloididae
Steinernematidae
Rhabditidae
Haemonchidae
Neodiplogasteridae
Onchocercidae
Ascarididae
Plectidae
Dorylaimidae
Qudsianematidae
Mononchidae
Nematoda
Trichinellidae
Prismatolaimidae
Hypsibiidae
Ramazzottiidae
Isohypsibiidae
Macrobiotidae
Tardigrada
Schistosomatidae
Opisthorchiidae
Hymenolepididae
Taeniidae
Brachionidae
Adinetidae
Ostreidae
Pteriidae
Pectinidae
Mytilidae
Veneridae
Vesicomyidae
Solecurtidae
Conidae
Ampullariidae
Achatinidae
Peltospiridae
Octopodidae
Lineidae
Enchytraeidae
Lumbricidae
Lumbricina
Megascolecidae
Spionidae
Priapulidae
Toxopneustidae
Asteriidae
0.2

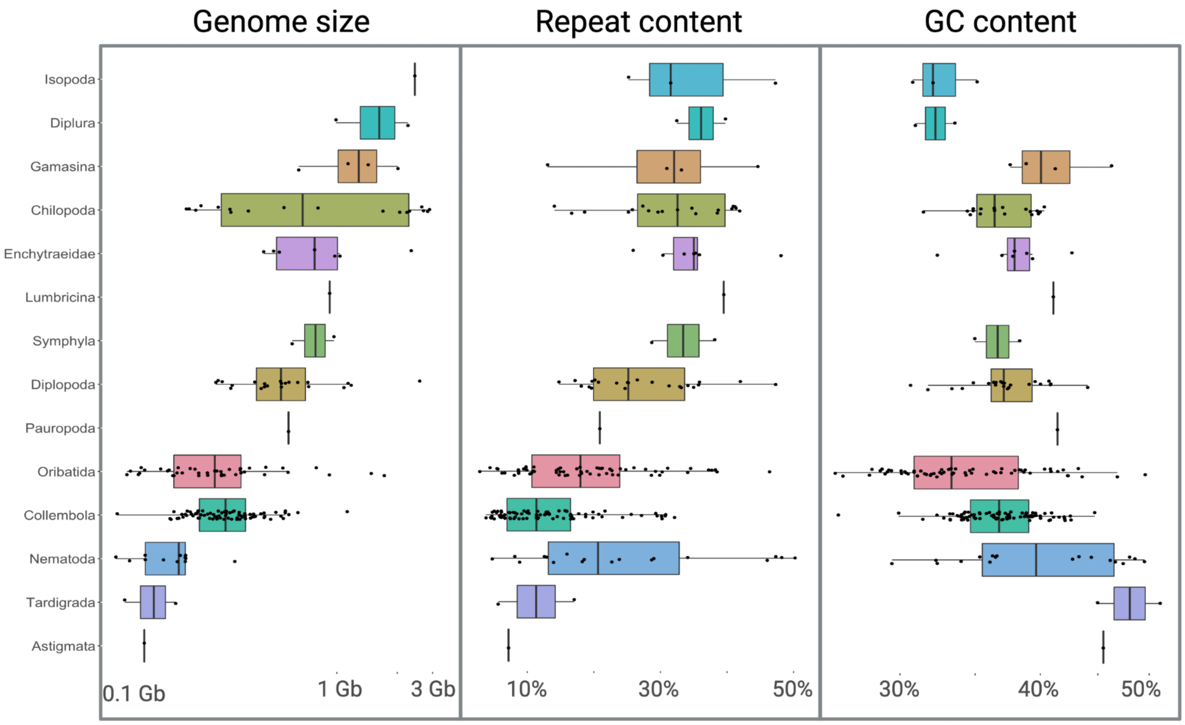

Supp. Fig. 2. Variation in soil invertebrate genome properties. Center line: median; box limits: upper and lower quartiles; whiskers: 1.5x interquartile range; points: outliers.

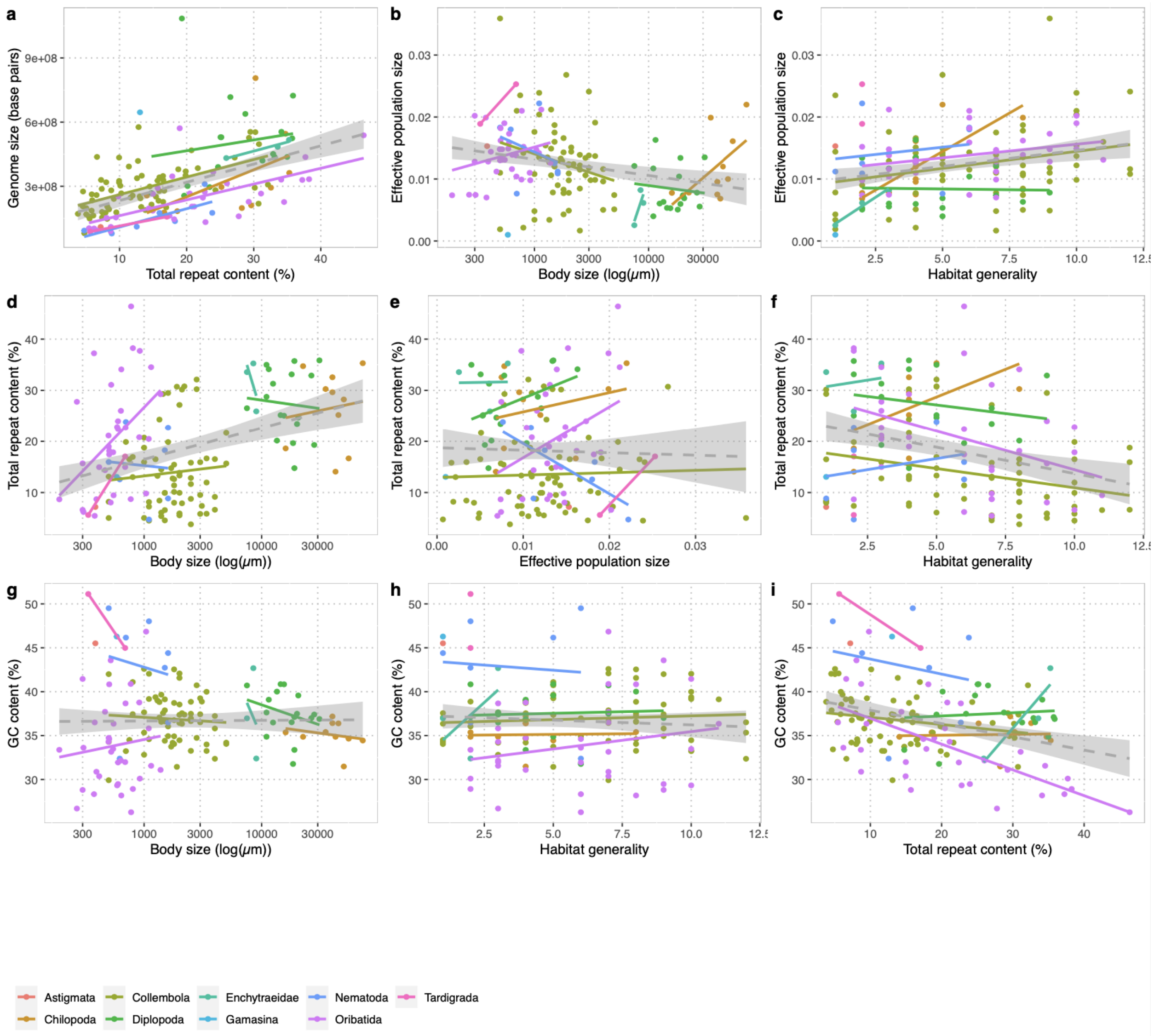

Supp. Fig. 3. Strength and direction of relationships among genome size, repeat content, GC content, and their ecological drivers vary among higher taxa of invertebrates. Dark grey dashed line represents a linear trendline for the entire regression, with light grey areas marking standard error. Colored lines represent regression trendlines of individual taxonomic groups, as noted in the figure legend. Statistical support of linear regressions: (a) $F = 54.94$, $df = 141$, $p < 0.001$; (b) $F = 11.19$, $df = 137$, $p = 0.001$; (c) $F = 10.32$, $df = 137$, $p = 0.002$; (d) $F = 22.47$, $df = 137$, $p < 0.001$; (e) $F = 0.11$, $df = 137$, $p = 0.74$; (f) $F = 13.58$, $df = 137$, $p < 0.001$; (g) $F = 0.01$, $df = 137$, $p = 0.91$; (h) $F = 0.69$, $df = 137$, $p = 0.41$; $F = 18.38$, $df = 137$, $p < 0.001$.
