# Supplementary material for: The MetaInvert soil invertebrate genome resource provides insights into below-ground biodiversity and evolution
Source: Commun Biol. 2023 Dec 8;6:1241. doi: 10.1038/s42003-023-05621-4 (PMC10709333; doi:10.1038/s42003-023-05621-4)
Supplement: Supplementary file 3 — Description of Additional Supplementary Files [file 42003_2023_5621_MOESM3_ESM.pdf]

Description of Additional Supplementary Files

File name: Supplementary Data 1
Description: Description of soil invertebrate samples and genomes. Includes collection data, genome statistics, NCBI accession numbers.

File name: Supplementary Data 2
Description: Kraken assignment data and R script behind Figure 3 in the paper.

File name: Supplementary Data 3
Description: Gene ontology enrichment results of metazoan core orthologs missing from oribatid mite and sprintail genomes.

File name: Supplementary Data 4
Description: The source data behind Figure 5c-d in the paper.

File name: Supplementary Data 5
Description: Summary of soil invertebrate collection methods.

File name: Supplementary Data 6
Description: List of benchmarking universal single copy orthologs used for phylogenetics.

File name: Supplementary Data 7
Description: Metazoan species used for computing the list of core metazoan genes.

File name: Supplementary Data 8
Description: Reference species for ortholog searches in springtails, nematodes, chilopods and diplopods, mites, tardigrades and enchytraeids.
