# Supplementary material for: The MetaInvert soil invertebrate genome resource provides insights into below-ground biodiversity and evolution
Source: Commun Biol. 2023 Dec 8;6:1241. doi: 10.1038/s42003-023-05621-4 (PMC10709333; doi:10.1038/s42003-023-05621-4)
Supplement: Supplementary file 7 — Reporting Summary [file 42003_2023_5621_MOESM7_ESM.pdf]

nature portfolio

Corresponding author(s): Miklós Bálint

Last updated by author(s): 30.7.2023

Reporting Summary

Nature Portfolio wishes to improve the reproducibility of the work that we publish. This form provides structure for consistency and transparency in reporting. For further information on Nature Portfolio policies, see our Editorial Policies and the Editorial Policy Checklist.

Statistics

For all statistical analyses, confirm that the following items are present in the figure legend, table legend, main text, or Methods section.

n/a	Confirmed	
☐	☒	The exact sample size (*n*) for each experimental group/condition, given as a discrete number and unit of measurement
☐	☒	A statement on whether measurements were taken from distinct samples or whether the same sample was measured repeatedly
☐	☒	The statistical test(s) used AND whether they are one- or two-sided<br>*Only common tests should be described solely by name; describe more complex techniques in the Methods section.*
☐	☒	A description of all covariates tested
☐	☒	A description of any assumptions or corrections, such as tests of normality and adjustment for multiple comparisons
☐	☒	A full description of the statistical parameters including central tendency (e.g. means) or other basic estimates (e.g. regression coefficient) AND variation (e.g. standard deviation) or associated estimates of uncertainty (e.g. confidence intervals)
☐	☒	For null hypothesis testing, the test statistic (e.g. *F*, *t*, *r*) with confidence intervals, effect sizes, degrees of freedom and *P* value noted<br>*Give P values as exact values whenever suitable.*
☒	☐	For Bayesian analysis, information on the choice of priors and Markov chain Monte Carlo settings
☒	☐	For hierarchical and complex designs, identification of the appropriate level for tests and full reporting of outcomes
☐	☒	Estimates of effect sizes (e.g. Cohen's *d*, Pearson's *r*), indicating how they were calculated

Our web collection on statistics for biologists contains articles on many of the points above.

Software and code

Policy information about availability of computer code

Data collection	No software was used for data collection.
Data analysis	All used software and versions are described in the Methods.

For manuscripts utilizing custom algorithms or software that are central to the research but not yet described in published literature, software must be made available to editors and reviewers. We strongly encourage code deposition in a community repository (e.g. GitHub). See the Nature Portfolio guidelines for submitting code & software for further information.

Data

Policy information about availability of data

All manuscripts must include a data availability statement. This statement should provide the following information, where applicable:

- Accession codes, unique identifiers, or web links for publicly available datasets
- A description of any restrictions on data availability
- For clinical datasets or third party data, please ensure that the statement adheres to our policy

Vouchers are deposited in the collections of the Senckenberg Museum of Natural History Görlitz (SMNG), Germany. Raw sequence files and draft assemblies will be accessible through the ENA/NCBI project PRJNA758215 NCBI accession codes for individual samples are listed in Supp. Table 1. 28S and COI barcodes are publicly available at dx.doi.org/10.5883/DS-TBGMI. Genome metadata can be accessed at the Genomes on a Tree (https://goat.genomehubs.org/projects/METAINVERT). Repeat elements can be accessed in the Dfam database (https://www.dfam.org/). No custom code or mathematical algorithms are central for the conclusions of the

paper.

Research involving human participants, their data, or biological material

Policy information about studies with human participants or human data. See also policy information about sex, gender (identity/presentation), and sexual orientation and race, ethnicity and racism.

Reporting on sex and gender	No human participants were involved in the research.
Reporting on race, ethnicity, or other socially relevant groupings	na
Population characteristics	na
Recruitment	na
Ethics oversight	na

Note that full information on the approval of the study protocol must also be provided in the manuscript.

Field-specific reporting

Please select the one below that is the best fit for your research. If you are not sure, read the appropriate sections before making your selection.

☐ Life sciences ☐ Behavioural & social sciences ☒ Ecological, evolutionary & environmental sciences

For a reference copy of the document with all sections, see nature.com/documents/nr-reporting-summary-flat.pdf

Ecological, evolutionary & environmental sciences study design

All studies must disclose on these points even when the disclosure is negative.

Study description	We sequenced and assembled the genomes of 232 species, representing 14 common soil invertebrate groups. We used the genomes to re-analyse the taxonomic assignment of metatranscriptomic sequences generated from soil environmental RNA. We tested generality of several hypotheses across soil invertebrate groups with structural equation models, parametrized with genome properties and ecological traits. We explored whether shared gene loss might be related to repeated adaptations of phylogenetically distant metazoans to soil conditions.
Research sample	Samples are soil invertebrate individuals or cultures from which genome sequences were generated. Each of these samples is a representative individual or a culture of a taxonomically accepted soil invertebrate species, described in details in Supp. Table 1.
Sampling strategy	Specimens were collected on the field or cultured by taxonomic experts of the respective groups. Sampling was done with specific methods that target the collection of these species for taxonomic purposes.
Data collection	Data collection is described in the Methods. Specific data collection and generation steps were done by the following co-authors: genome assembly - C. Schneider, quality assembly with BUSCO genes - J. Romahn, metatranscriptomic assignments - R. Veres, M. Balint, A. Schmidt, D. Merges; phylogeny - J. Romahn; genome size estimation - M. Pfenninger, C. Schneider, G. Collins; effective population size estimation - M. Pfenninger; repeat annotation - L. Bostjancic, O. Lecompte, C. Rutz; ecological trait data collection - G. Collins, R. Lehmitz, C. Schneider, P. Decker, R. Schmelz, J. Römbke, K. Hohberg, A. Christian, U. Burkhardt; structural equation models - G. Collins; gene loss analyses - H. Mülbaier, I. Ebersberger, G. Collins.
Timing and spatial scale	The temporal and spatial scales are not relevant for the sampling. Sample locations and dates are stated in Supp. Table 1.
Data exclusions	The exclusion of individual samples was done based on pre-defined critreria which are presented in the methods.
Reproducibility	No experiments were performed.
Randomization	Samples were allocated into groups based on taxonomics identity, whenever comparison of taxonomic groups were relevant for the study question.
Blinding	Since we aimed to generate a broad genome resource covering multiple distinct groups (families, genera etc.) of soil invertebrates, blinding is not relevant for the study.

Did the study involve field work? ☒ Yes ☐ No

Field work, collection and transport

Field conditions	Sampling time and location was decided by the taxonomic experts based on experience with the soil invertebrate groups. Environmental parameters (rainfall, temperature) are not relevant for the sampling.
Location	Sample locations and dates are stated in Supp. Table 1.
Access & import/export	Sample acquisition was done along the requirements of the Nagoya Protocol on Access to Genetic Resources and the Fair and Equitable Sharing of Benefits Arising from their Utilization (ABS) to the Convention on Biological Diversity.
Disturbance	Samples were obtained by minimally invasive approaches, e.g. from small, 1-5 cm diamater soil cores, nets, or hand picking.

Reporting for specific materials, systems and methods

We require information from authors about some types of materials, experimental systems and methods used in many studies. Here, indicate whether each material, system or method listed is relevant to your study. If you are not sure if a list item applies to your research, read the appropriate section before selecting a response.

Materials & experimental systems

n/a	Involved in the study	
☒	☐	Antibodies
☒	☐	Eukaryotic cell lines
☒	☐	Palaeontology and archaeology
☐	☒	Animals and other organisms
☒	☐	Clinical data
☒	☐	Dual use research of concern
☐	☒	Plants

Methods

n/a	Involved in the study	
☒	☐	ChIP-seq
☒	☐	Flow cytometry
☒	☐	MRI-based neuroimaging

Animals and other research organisms

Policy information about studies involving animals; ARRIVE guidelines recommended for reporting animal research, and Sex and Gender in Research

Laboratory animals	The study did not involve laboratory animals.
Wild animals	Soil invertebrates were collected in the wild as described in the Methods or in Supp. Table 1. They were either sampled on the field and euthanized immediately in ethanol, or they were transported alive to the laboratory in the soil matrix where they were extracted and euthanized according to practices dealing with the respective group. Several samples were available as living cultures in the labs of one or more of the co-authors. Specimens from these cultures were euthanized with ethanol before DNA extraction.
Reporting on sex	Sex is not relevant for the study, although sexuality and parthenogenesis are. This is stated in Supp. Table 1.
Field-collected samples	The most important consideration for field-collected samples was to ensure proper DNA preservation until DNA extraction. Specimens were mostly preserved in 96% ethanol.
Ethics oversight	No animal experimentation was performed during the research. Only the minimum sufficient numbers of specimens were used for genome sequencing.

Note that full information on the approval of the study protocol must also be provided in the manuscript.
