# Supplementary material for: The MetaInvert soil invertebrate genome resource provides insights into below-ground biodiversity and evolution
Source: Commun Biol. 2023 Dec 8;6:1241. doi: 10.1038/s42003-023-05621-4 (PMC10709333; doi:10.1038/s42003-023-05621-4)
Supplement: Supplementary file 6 — Supplementary Data 4 [file metazoa_core_genes_in_meta_invertebrates.pdf › Supplementary Data Fig5cd/metazoa_core_genes_in_meta_invertebrates.pdf]

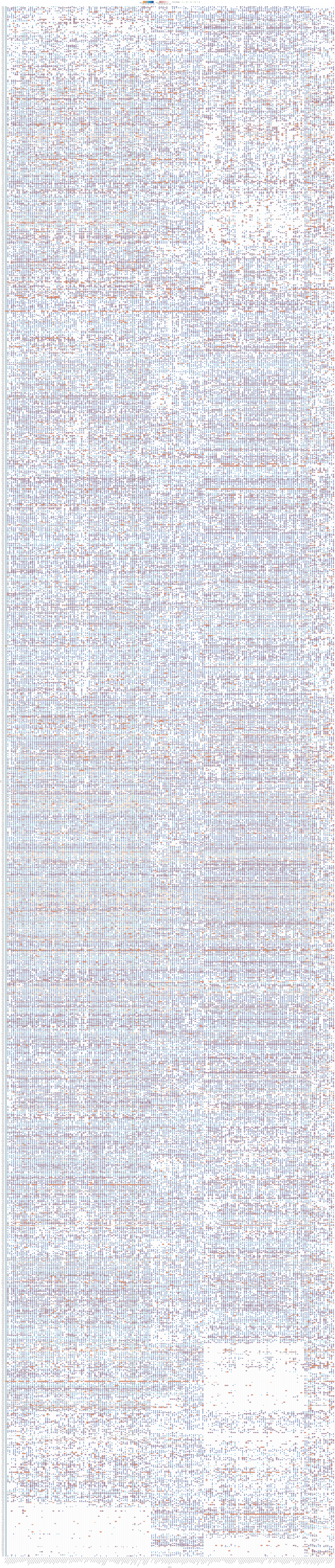
FAS_F
0.00 0.25 0.50 0.75 1.00
FAS_B
0.00 0.25 0.50 0.75 1.00
# of co-orthologs
5 10 15 20 25
